# Validation of Pinnacle treatment planning system for use with Novalis delivery unit

**DOI:** 10.1120/jacmp.v11i3.3240

**Published:** 2010-06-15

**Authors:** Vladimir Feygelman, Dylan Hunt, Luke Walker, Richard Mueller, Mary Lou Demarco, Thomas Dilling, Craig Stevens, Geoffrey Zhang

**Affiliations:** ^1^ Division of Radiation Oncology H. Lee Moffitt Cancer Center Tampa Florida 33612 USA

**Keywords:** accelerator commissioning, Novalis, intensity‐modulated radiation therapy, volumetric intensity modulated arc therapy, diode array dosimeter

## Abstract

For an institution that already owns the licenses, it is economically advantageous and technically feasible to use Pinnacle TPS (Philips Radiation Oncology Systems, Fitchburg, WI) with the BrainLab Novalis delivery system (BrainLAB A.G., Heimstetten, Germany). This takes advantage of the improved accuracy of the convolution algorithm in the presence of heterogeneities compared with the pencil beam calculation, which is particularly significant for lung SBRT treatments. The reference patient positioning DRRs still have to be generated by the BrainLab software from the CT images and isocenter coordinates transferred from Pinnacle. We validated this process with the end‐to‐end hidden target test, which showed an isocenter positioning error within one standard deviation from the previously established mean value. The Novalis treatment table attenuation is substantial (up to 6.2% for a beam directed straight up and up to 8.4% for oblique incidence) and has to be accounted for in calculations. A simple single‐contour treatment table model was developed, resulting in mean differences between the measured and calculated attenuation factors of 0.0%–0.2%, depending on the field size. The maximum difference for a single incidence angle is 1.1%. The BrainLab micro‐MLC (mMLC) leaf tip, although not geometrically round, can be represented in Pinnacle by an arch with satisfactory dosimetric accuracy. Subsequently, step‐and‐shoot (direct machine parameter optimization) IMRT dosimetric agreement is excellent. VMAT (called “SmartArc” in Pinnacle) treatments with constant gantry speed and dose rate are feasible without any modifications to the accelerator. Due to the 3 mm‐wide mMLC leaves, the use of a 2 mm calculation grid is recommended. When dual arcs are used for the more complex cases, the overall dosimetric agreement for the SmartArc plans compares favorably with the previously reported results for other implementations of VMAT: γ(3%,3mm) for absolute dose obtained with the biplanar diode array passing rates above 97% with the mean of 98.6%. However, a larger than expected dose error with the single‐arc plans, confined predominantly to the isocenter region, requires further investigation

PACS numbers: 87.55Qr, 87.56Nk

## I. INTRODUCTION

The BrainLab Novalis radiotherapy unit (Novalis system, BrainLAB A.G., Heimstetten, Germany) is a complete radiation therapy delivery system, including treatment planning, image guidance and delivery modules. It has many attractive features such as a high definition micro‐MLC (mMLC),^(^ ^1^ ^)^ robotic table with six degrees of freedom,^(^ ^2^ ^)^ and a robust combination of a flat‐panel X‐ray stereoscopic imaging system and an infrared tracking system for patient positioning.^(^ ^3^ ^)^ Several researches have reported overall submillimeter targeting accuracy of the Novalis delivery process in phantoms.^(^ ^3^ ^–^ ^5^ ^)^ The Novalis system is widely used for stereotactic body radiation therapy (SBRT) in a variety of sites.^(^ ^6^ ^)^ However, a major limitation of the BrainLab treatment planning system in use at our institution (iPlan RT Dose v. 3.0.2) is the pencil beam dose calculation algorithm. It is well established that dose calculations with pencil beam algorithms lose accuracy in the presence of low‐density inhomogeneities typical, for example, in lung treatments.^(^ ^7^ ^)^

Although excellent clinical results were reported for lung SBRT planned with the iPlan pencil beam algorithm,^(^ ^8^ ^)^ it is ultimately preferred to employ a more accurate dose calculation engine. The Pinnacle treatment planning system (Philips Radiation Oncology Systems, Fitchburg, WI) employs a collapsed cone convolution (CCC) algorithm.^(^ ^9^ ^,^ ^10^ ^)^ This method is currently regarded as one of the better practical options for dose calculation in lung.^(^ ^7^ ^)^

This paper describes validation of Pinnacle as a treatment planning engine for the Novalis image guidance and delivery system. Although the commissioning process in many respects is the same as for a standard Varian linear accelerator,^(^ ^11^ ^)^ a number of issues are unique to the configuration in question. The Novalis image guidance system (ExacTrac) does not accept digitally reconstructed radiographs (DRRs) generated by an external TPS. The process of performing the dose planning with Pinnacle and generating the DRRs with ExacTrac had to be validated. A rather high attenuation by the robotic couch table top was first reported by Njeh et al.^(^ ^12^ ^)^ Since Pinnacle currently lacks an explicit built‐in routine to model the tabletop, a practical method to account for the couch attenuation had to be developed. The leaf‐end shape of the Novalis mMLC differs from the standard Varian Millennium MLC, and the optimal model parameters to best describe it in Pinnacle had to be determined. Finally, as the Pinnacle volumetric modulated arc therapy (VMAT)^(^ ^13^ ^–^ ^15^ ^)^ module (called SmartArc^(^ ^16^ ^)^) became available, it was instructive to study the feasibility of its implementation with the Novalis system in addition to standard direct machine parameter optimization (DMPO)^(^ ^17^ ^)^ IMRT. While it was recently demonstrated that SmartArc planning can be done for a standard Varian accelerator with a constant gantry speed and dose rate,^(^ ^18^ ^)^ the effects of differences in the mMLC design compared to the Millennium MLC needed to be explored.

## II. MATERIALS AND METHODS

### A. Radiation delivery system

The linear accelerator is a modified 6 MV Varian Clinac 600C (Varian Medical Systems, Palo Alto, CA). The usable field size is limited to $9.8\;\times \;9.8\;{\text{cm}}^{2}$. Because of this small field size, the flattening filter is shortened to provide higher dose rate (up to 800 MU/min). The unit is equipped with a permanently mounted mMLC.^(^ ^19^ ^)^ It has 26 leaf pairs moving along the Y‐jaw direction. The leaves have different widths, the thinnest ones in the center projecting to the isocenter at 3 mm. The leaf can span the entire 9.8 cm field length. The published maximum leaf speed is 1.5 cm/sec.^(^ ^1^ ^)^

**Figure 1:**
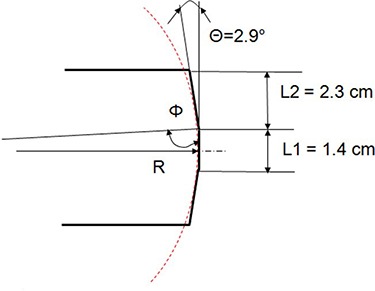
The mMLC leaf sketch with the circle tangent to all three edges. The X‐axis scale is expanded for clarity.

Because of the limited field size, the leaf end shape is less complex than in the standard rounded‐end Varian MLC^(^ ^20^ ^)^ that has to cover a span of ±20 cm. The most detailed diagram of the mMLC leaves was provided by Belec et al.^(^ ^21^ ^)^ (Fig. 1). Cosgrove et al.^(^ ^1^ ^)^ explained how the leaves are focused towards the target; the central ray is parallel to the vertical edge with the leaf at isocenter, and to the slanted edges when the leaf is extended to −5 cm or retracted to +5 cm. For the edges to line up with the central ray under these conditions, the slant angle Θ in Fig. 1 should be equal to *arctan*(5/100), or 2.9°. This contradicts the 1.8° value presented by Belec et al.^(^ ^21^ ^)^ but compares favorably to approximately 3° quoted by Cosgrove et al.^(^ ^1^ ^)^

### B. Record and verify system

The current clinical version of the Impac Mosaiq record and verify (R&V) system was used (v.1.60, Impac Medical Systems, Sunnyvale, CA). The machine characterization file for the Novalis system already included an activated entry for the gantry‐angle indexed arc treatment, corresponding to the BrainLab iPlan specifications. The corresponding MLC control files are called arc‐dynamic. It is important to distinguish this from the monitor unit indexed MLC files (dose‐dynamic) necessary for the VMAT delivery with variable gantry speed. Treatment plans were transferred from the TPS to R&V via DICOM RT.

### C. Treatment planning system

#### C.1 Beam model – Leaf‐end

We used Pinnacle v. 9.0, which was very recently released. The general data collection and commissioning of the TPS largely followed Cadman et al.^(^ ^11^ ^)^ Because of the limited field size, all PDDs and cross‐beam profiles in the water tank were acquired with the shielded Scanditronix Photon Field diode (PFD, IBA Dosimetry, Schwarzenbruck, Germany). The active volume is approximately 1.8 mm in diameter. The use of this dosimeter for Pinnacle commissioning was previously thoroughly validated by comparison with ion chamber and film.^(^ ^11^ ^)^ The MLC transmission was estimated from the best fit of the calculated and diode‐measured cross‐beam profiles just outside the MLC‐defined fields.

The leaf end shape is represented by an arc of a circle in the Pinnacle beam model.^(^ ^11^ ^,^ ^22^ ^)^ As a starting point, the radius *R* of the circle tangent to all three edges of the leaf (Fig. 1) can be found from the following equation:

(1) $$R=\frac{L_{1}}{2}\text{tan}(\Phi )$$

where

(2) $$\Phi =\frac{180^{{}^{\circ}}-\Theta }{2}$$

As pointed out by Williams and Metcalfe,^(^ ^22^ ^)^ the dose profiles through the junction of the two abutting MLC‐defined fields are a very sensitive method to asses the dosimetric accuracy of the leaf‐end model.

To that end, two $3\times 3\;{\text{cm}}^{2}$ MLC‐defined fields were slowly scanned in the water tank along the leaf movement direction (Y) at the 10 cm depth. Each field was offset by 1.5 cm from the central axis, so they abutted at the isocenter. The scan line was offset from the central axis by 1.5 mm in the X direction to avoid the intra‐leaf leakage. The resulting scans were converted to absolute dose by comparison to the reference field static reading. The scans were numerically added, averaged and compared to the corresponding TPS absolute dose profiles calculated on the 1 mm grid. The radius *R* was adjusted in the model to obtain the best dosimetric agreement.

#### C.2 Beam model – MLC transmission

To confirm the modeling accuracy of the small MLC‐defined fields, a bar pattern^(^ ^11^ ^)^ was scanned with the diode in the direction orthogonal to the leaf movement. The diode reading was again normalized to the reference field to produce absolute dose profiles. The smallest opening was four‐leaf wide (1.2 cm at the isocenter).

Finally, a typical set of point‐dose measurements for a variety of open and MLC‐defined fields was performed with a microchamber in the $20\times 20\times 20\;{\text{cm}}^{3}$ Cube Plastic Water phantom (CIRS Inc, Norfolk, VA) for comparison with the TPS calculations.

#### C.3 Table attenuation

It was noticed that the BrainLab robotic couch introduces fairly significant attenuation in the beam – anywhere between 3% and 8%, depending on the incidence angle and method of measurement.^(^ ^12^ ^,^ ^23^ ^)^ The values quoted above were obtained with the standard Varian Clinac 6 MV beams. The high dose rate Novalis beam has a lower energy because of a modified flattening filter. The attenuation values had to be remeasured. This was done with an ion chamber placed at the center of the Cube Plastic water phantom. The gantry angle was varied in 10° increments. The values were averaged for the beam directions symmetrical with respect to vertical.

The modeling of the treatment table in Pinnacle was recently reported by Mihaylov et al.^(^ ^23^ ^)^ The table has a non‐homogeneous structure and its full representation requires a number of contours.^(^ ^23^ ^)^ Pinnacle currently does not have a built‐in facility to include the treatment table in the plan, as described for another TPS.^(^ ^24^ ^)^ We wanted to explore if the table can be adequately modeled as a single contour with an average density. The outer dimensions of the table were contoured and digitized into the TPS. The first approximation of the average density was derived from the published weighted densities of the carbon fiber shell and the filler foam.^(^ ^23^ ^)^ Then it was adjusted to best fit the measured attenuation data at different angles in 10° increment for the 5×5 and $10\times 10\;{\text{cm}}^{2}$ beams. The model was validated by comparing the measured and calculated dose for a $7\times 7\;{\text{cm}}^{2}$ beam sweeping the posterior aspect of the phantom in a 180° arc. Because the gantry cannot rotate through the 180° angle, the arc was delivered in two segments of 90° each. The table attenuation was included in all dose calculations used in this work.

### D. Targeting accuracy end‐to‐end test

After the plan is generated in Pinnacle, the patient CT and the plan isocenter coordinates are transferred to iPlan via DICOM RT. The posterior oblique patient alignment DRRs are then generated by the ExacTrac system based on this anatomical information. To validate this procedure, a global hidden target test with an anthropomorphic head phantom was performed. This test, as adopted at our institution, was described in detail previously.^(^ ^25^ ^)^ In brief, two orthogonal pieces of radiochromic film are inserted in the middle of a spherical target in the axial and coronal orientations. The phantom is scanned, planned, aligned and irradiated. The films are scanned with a flat bed scanner and the position of the optical density centroid is compared to the center of the target, which is intended to be positioned at the isocenter.

### E. Inverse planning

#### E.1 Structures and objectives

The same set of structures, dose objectives and measurement specifications was used for step‐and‐shoot IMRT and VMAT plans, as described in the AAPM TG119 report.^(^ ^26^ ^)^ In the approximate order of increasing plan complexity, the structure sets included a mock prostate, three stacked cylinders receiving different doses (multitarget), a mock head and neck, and a C‐shape with an avoidance core. The structures were projected onto the CT scan of the Plastic Water Cube phantom, as recommended in the TG119 report. The phantom design allows for easy chamber position changes in three orthogonal directions, facilitating point dose measurements in the target and avoidance structures. The stacked cylinders and mock head and neck volumes had to be modified to fit into the $10\times 10\;{\text{cm}}^{2}$ field with a reasonable dosimetric margin. All the cylinder lengths were reduced in the same proportion in the former case. For the latter, the PTV volume was shrunk in 3D.

#### E.2 DMPO planning

Beam arrangements described in the TG119 report were used for the step‐and‐shoot plans with minor modifications. The starting angle was offset from the vertical to avoid beam incidence directly along the biplanar diode array dosimeter detector boards.^(^ ^4^ ^)^ A single step‐and‐shoot (DMPO) plan was generated for each structure set.

#### E.3 SmartArc planning

Depending on the capabilities of the accelerator, SmartArc can generate rotational inverse plans with variable or constant gantry speed and dose rate.^(^ ^16^ ^)^ It was demonstrated recently that clinically feasible VMAT plans can be successfully delivered on a standard Varian linac in the constant gantry speed/dose rate mode.^(^ ^18^ ^)^ We took the same approach in this work, since the Novalis linac is not equipped with full VMAT capability.

A number of plans were generated to explore different compromises arising in the VMAT machine modeling and planning. The first issue is the leaf speed constraint. With the standard linac configuration, the VMAT dose modulation is already somewhat limited by the inability to vary either the gantry speed or dose rate during the delivery. Therefore, it is beneficial to use the maximum leaf speed consistent with good dosimetric accuracy and uninterrupted delivery. There are two settings that constrain the leaf speed in the SmartArc algorithm. One is straightforward – the maximum leaf speed. The other is more obscure. Both Otto^(^ ^14^ ^)^ and Bzdusek et al.^(^ ^13^ ^)^ have noted that it is necessary to constrain the amount of leaf motion per degree of gantry rotation ${\left(\text{dx}/d\Theta \right)}_{\max}$:

(3) $${\left(\frac{dx}{d\Theta }\right)}_{\text{max}}={\left(\frac{dx}{dt}\right)}_{\text{max}}/{\left(\frac{d\Theta }{dt}\right)}_{\text{max}}$$

where ${\left(\text{dx}/\text{dt}\right)}_{\max}$ is the maximum leaf speed in cm/sec, and ${\left(d\Theta /\text{dt}\right)}_{\max}$ is the maximum gantry rotational speed in deg/sec.

There is no accelerator specification limiting the MLC motion per degree per se. If the planned delivery time equals the actual one, Eq. (3) is redundant and reduces to the simple maximum leaf speed constraint. However, in the planning mode with the constant gantry speed and dose rate, the SmartArc algorithm allows the user to set the maximum allowed delivery time. The optimizer would then adjust the gantry speed and dose rate to approximately meet this requirement. When this arc‐dynamic plan is transferred to the linac controller, its software has no information about the planned delivery time and chooses the gantry speed and the dose rate to execute the plan as quickly as possible, constrained only by the maximum gantry speed, the highest available dose rate and the total monitor units. As a result, the plans optimized with the higher maximum delivery time settings are typically delivered faster than predicted by Pinnacle. Since the total leaf travel stays the same while the treatment time is reduced compared to the plan, the leaf speed has to be higher and may exceed the ${\left(\text{dx}/\text{dt}\right)}_{\max}$ constraint. In this case, Eq. (3) takes over in enforcing the leaf speed limit, since the total leaf travel and the gantry angular travel stay the same. We exploited this feature by setting the different values of ${\left(\text{dx}/d\Theta \right)}_{\max}$ and thus obtaining plans with different maximum leaf speeds. This is more convenient than changing the maximum leaf speed in the TPS machine editor because ${\left(\text{dx}/d\Theta \right)}_{\max}$ can be changed in the planning mode without recommissioning the Pinnacle machine.

The goal was to investigate the plan quality and dosimetric agreement for a set of SmartArc plans with different planned delivery times (and, consequently, leaf speeds). To reduce the number of variables, all plans were optimized with 2° control point (CP) spacing. This eliminates the small but noticeable additional errors introduced by the small‐arc approximation with the 4° spacing, particularly for more complex plans.^(^ ^27^ ^)^ Having a number of different plans for each structure sets also provides more robust statistics for the dosimetric agreement analysis.

#### E.4 Dynamic log (Dynalog) file analysis

Varian Dynalog files are known to be a robust method of analyzing the leaf position deviation from the prescription during beam delivery.^(^ ^28^ ^,^ ^29^ ^)^ They were recorded for a number of SmartArc plans and analyzed with Varian Argus v. 4.7 software. The available data include the average RMS deviations across all leaves, the maximum RMS deviation in that cohort, the 95% range on the error histogram, the maximum leaf deviation, and the leaf speed. In addition to the SmartArc plans, the Dynalog analysis was applied to the simple leaf‐speed test dynamic arc plan similar to that in the Varian acceptance test.^(^ ^30^ ^)^ It helped to establish the baseline for the expected leaf positioning accuracy for different leaf speeds, and also to find the maximum possible leaf speed. Leaf speed *v* is governed by the following equation:

(4) $$v=S\times \dot{D}/MU$$

where *S* is the total leaf travel in the plan (70 cm for BrainLab mMLC with the acceptance test plan used), *D* is the dose rate in monitor units per second, and *MU* is the total plan monitor units. By maintaining the constant arc angle and decreasing the MU, it is possible to push the leaves to their maximum attainable speed.

#### E.5 Experimental dosimetric agreement for inverse planning

Point doses were measured with the microchamber in the Cube phantom for the target and avoidance structures. The percent errors were normalized to the prescription dose.^(^ ^26^ ^)^

For the dose distribution, we used the biplanar diode dosimeter (Delta4, ScandiDos AB, Uppsala, Sweden). This device was validated by various groups for step‐and‐shoot IMRT,^(^ ^4^ ^,^ ^31^ ^,^ ^32^ ^)^ VMAT^(^ ^31^ ^)^ and Helical TomoTherapy.^(^ ^33^ ^)^ The dosimeter consists of a 22 cm diameter PMMA phantom with two orthogonal detector boards. The diode detectors are spaced by 5 mm in the central $6\times 6\;{\text{cm}}^{2}$ area of each board and by 10 mm in the remainder of the $20\times 20\;{\text{cm}}^{2}$ active area. The reference dose is calculated by the TPS on the phantom CT scan and is transferred to the Delta4 software by DICOM RT. The dosimeter registers absolute dose and has a standard suite of IMRT dose‐comparison software tools. It has been repeatedly shown that the dosimeter accuracy and reproducibility is satisfactory and that gamma analysis^(^ ^34^ ^)^ passing rates in excess of 95% are routinely achievable for a well‐commissioned TPS for at least γ(3%,3mm). The Delta4 dosimeter is particularly suitable for the arc delivery verification,^(^ ^27^ ^,^ ^31^ ^,^ ^33^ ^,^ ^35^ ^)^ where the field‐by‐field analysis with a planar detector array^(^ ^26^ ^)^ is not possible. We used the γ(3%,3mm) criterion as the primary method of comparing the measured and reference dose distributions. The dose‐error threshold is global and represents 3% of the prescription dose. All detectors receiving more than 10% of the prescription dose are included in the analysis. The short‐term measurement reproducibility was confirmed by repeating the delivery of two different plans. The effects of the calculation grid size and maximum leaf speed on dosimetric accuracy were studied.

## III. RESULTS

### A. TPS beam model

#### A.1 Leaf‐end radius

**Figure 2:**
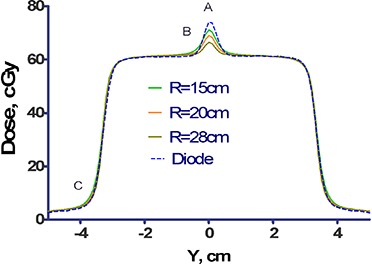
Dose profiles through abutting MLC fields. The dose profile consists of the sum of two $3\times 3\;{\text{cm}}^{2}$ MLC fields, each offset by 1.5 cm from the central axis, and measured at a depth of 10 cm and with an offset of 1.5 mm in the X direction. Also plotted are the corresponding profiles calculated by the TPS for three selected values of the leaf‐radius, R=15, 20, and 28 cm.

Substituting the leaf dimensions from Fig. 1 into Eq. (1), the radius of the circle tangent to all three edges of the leaf is approximately 28 cm. As shown in Fig. 2, the magnitude and location of the dosimetric disagreement vary depending on the choice of the radius *R*. The RMS differences between the measured and calculated doses were 2.0, 1.8, and 1.6% for R=28, 15, and 20 cm, respectively. The compromise value of 20 cm was chosen for the leaf tip radius in the beam model. It results in an 8% error at the junction, but reduces the error at the shoulder to 0.8%.

#### A.2 MLC transmission and static field dosimetry

The best fit MLC transmission determined by the Pinnacle automodeling script was 0.9%. This is essentially the same value as 0.93±0.05% measured recently by Garcia‐Garduno et al.^(^ ^36^ ^)^ In the same work, the interleaf leakage was measured at 1.08±0.08%. Therefore we used the standard interleaf leakage value of 1%.

The resulting set of PDDs and cross‐beam profiles deviated from measurement by no more than 1%/1mm, when the error was normalized to the central axis dose as recommended by the AAPM TG53 report.^(^ ^37^ ^)^ The bar pattern^(^ ^11^ ^)^ results are similar. A set of 26 point dose measurements for a number of jaw‐ and MLC‐defined fields in a phantom resulted in an average error of −0.2±0.6%. The mean is not statistically different from 0 (t‐test p=0.1).

#### A.3 Table attenuation

The measured table attenuation was about 6% with the gantry at 180° (Varian IEC) and it reached the maximum of 8% with the gantry at 130°. According to the published data,^(^ ^23^ ^)^ a radiation beam at a normal incidence to the Novalis table will traverse 0.4 cm of the carbon fiber shell with the density of $0.7\;g/{\text{cm}}^{3}$ and 4.6 cm of foam with the density of $0.1\;g/{\text{cm}}^{3}$. This results in the calculated average weighted table density of $0.15\;g/{\text{cm}}^{3}$. Based on our CT measurements, the average density was $0.16\;g/{\text{cm}}^{3}$. The average density best fitting the attenuation data was found to be $0.23\;g/{\text{cm}}^{3}$. For the $10\times 10\;{\text{cm}}^{2}$ field, the mean difference across all nine gantry angles is 0.0±0.6% (range from −1.1 to 0.7%). For the $5\times 5\;{\text{cm}}^{2}$ field it was −0.2±0.5% (range from −0.8 to 0.5%). The measured isocenter dose for a 180° posterior arc with the $7\times 7\;{\text{cm}}^{2}$ collimator opening was within 0.6% of that predicted by Pinnacle.

### B. Targeting accuracy end‐to‐end test

The 3D displacement of the dose distribution center of mass from the geometrical target center was measured at 1.07 mm. This value is within the 95% confidence interval (CI) previously established for the same unit with the native software (mean 0.83±0.40 mm, 95% CI 0.52 to 1.10 mm). The isocenter and CT transfer from Pinnacle to ExacTrac do not degrade targeting accuracy compared to iPlan.

### C. Inverse planning dosimetry

#### C.1 DMPO planning results and dosimetric agreement

**Figure 3:**
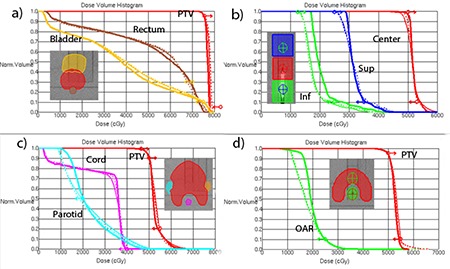
Representative DVHs: a) Mock Prostate; b) Multi‐Target; c) Mock H&N (SmartArc planned with two arcs); d) C‐shape (SmartArc with two arcs). Thick solid line=DMPO, thin solid=SmartArc with MLC constrained to 0.22 cm/deg, dashed=0.33 cm/deg.

The DMPO DVHs are represented by thick lines in Fig. 3. The corresponding dosimetric agreement parameters are summarized in Table 1. While the γ(3%,3mm) is most often used clinically to quantify the dosimetric accuracy of the IMRT systems,^(^ ^26^ ^,^ ^38^ ^)^ the γ(2%,2mm) is presented here when the 3%/3mm criterion is not sensitive enough. The overall mean ion chamber point dose error is 0.5±0.5%. The γ(3%,3mm) passing rates are uniformly high (≥97.9%). The γ(2%,2mm) criterion provides the passing rate spread from 86% to 98%.

**Table 1:** Dosimetric results for the DMPO plans: ion chamber (IC) point dose errors and Delta4 dose distribution gamma analysis.

*Plan*	*IC error PTV, %*	*IC error OAR, %*	%γ(3%,3mm)≤1.0	%γ(2%,2mm)≤1.0
Mock Prostate	0.0	0.6	100	98.3
Multi‐Target	1.6	0.5	97.9	86.2
Mock H&N	0.3	0.1	99.0	89.5
C‐shape	0.3	0.4	99.2	95.6

#### C.2 SmartArc – planning results and dosimetric agreement with different leaf speeds

Dose‐volume histograms for representative plans for each structure set are shown in Fig. 3. The thin solid lines correspond to the 0.22 cm/deg (1.0 cm/sec) MLC constraint, and dashed ones to the 0.33 cm/deg (1.5 cm/sec) one. The only difference in terms of meeting the dose‐volume objectives is a slight improvement in the PTV coverage for the multi‐target case with the higher leaf speed (Fig. 3(b)).

The gamma analysis results for the two groups of plans are presented in Table 2. If anything, there is a slight improvement in dosimetric agreement with the MLC motion constrained to 0.33 cm/deg. The differences are small and, in the subsequent discussion, the plans with both MLC constraint levels are grouped together to improve the statistical power of the analysis.

**Table 2:** Gamma analysis results for the plans with the MLC motion constrained to 0.22 cm/deg vs. 0.33 cm/deg.

*Plan*	*N*	*MLC constrained 0.22 cm/deg*		*MLC constrained 0.33 cm/deg*	
		Mean %γ(3%,3mm)≤1.0	*Range*	Mean %γ(3%,3mm)≤1.0	*Range*
Mock Prostate	2	100	‐	100	‐
Multi‐Target	3	97.3	96.3–97.9	97.4	96.3–98.2
Mock H&N	5	95.9	93.3–97.7	97.0	95.2–99.7
C‐shape	4	95.0	90.2–98.9	95.1	90.2–99.0

#### C.3 SmartArc – effect of calculation grid size on dosimetric agreement

The gamma analysis statistics for the same plans but with the final dose calculated on the 2 vs. 3 mm grid are presented in Table 3. The mean passing rates increase consistently as the calculation grid size is reduced. More importantly, the lowest passing rate improves by 3–5 percentage points for all the plan types but the simplest (prostate). For the prostate, the γ(3%,3mm) criterion is not sensitive enough. With the γ(2%,2mm) test, the average passing rate increases from 96.9% to 98.4%, with the ranges changing from 96.4%–97.4% to 97.6%–99.1%. The γ(3%,3mm) passing rate is above 90% for all the plans calculated on the 2 mm grid. Since a clear dosimetric accuracy advantage is seen with the 2 mm calculation grid, it is used exclusively in the subsequent discussion of the SmartArc dosimetry.

**Table 3:** Gamma analysis for SmartArc plans calculated on 3 vs. 2 mm grid.

*Plan*	*N*	*3 mm Grid*		*2 mm Grid*	
		Mean %γ(3%,3mm)≤1.0	*Range*	Mean %γ(3%,3mm)≤1.0	*Range*
Mock Prostate	4	99.9	99.6–100	100	‐
Multi‐Target	6	94.3	93.2–96.0	97.4	96.3–98.2
Mock H&N	5	94.2	89.5–99.5	97.0	95.2–99.7
C‐shape	7	93.8	85.9–98.7	95.7	90.2–99.0

#### C.4 Dynalog file analysis and maximum leaf speed

As a first step, a leaf speed test was executed with three different total MUs, selected to produce maximum leaf speeds of 1.0, 1.5, and 2.0 cm/sec according to Eq. (4). The 1.0 cm/sec value is used in the Varian acceptance test.^(^ ^30^ ^)^ On the other hand, the published maximum leaf speed is 1.5 cm/sec.^(^ ^19^ ^)^ The 2.0 cm/sec speed was expected to be close to the maximum achievable. The results are presented in Table 4. The larger deviations of the two leaf banks are reported. The maximum attainable leaf speed was approximately 1.8 cm/sec. The Dynalog results with this leaf speed do not meet the Varian dynamic arc test specifications (95% of the counts with less than 0.35 cm error), and it was excluded from further consideration. Since the manufacturer's specifications were easily met with both 1.0 and 1.5 cm/sec leaf speed values, the plans with the two corresponding MLC constraints (0.22 and 0.33 cm/deg from Eq. (3)) were developed and compared. The overall Dynalog statistics are presented in Table 5. The difference between both average and maximum RMS leaf position deviations for the 0.22 and 0.33 cm/deg constrained plans (averaged across 18 plans for each group) is statistically significant (t‐test p≤ 0.0003).

**Table 4:** Dynalog results for the dynamic arc leaf speed test.

*Leaf Speed, cm/s*	*Average RMS Error, cm*	*Max RMS Error, cm*	*% Errors* <0.35 cm	*Max Leaf Deviation*
1.0	0.052	0.053	100	0.108
1.5	0.106	0.157	99.8	−0.483
1.8	0.239	0.341	88.2	0.842

**Table 5:** Dynalog analysis results across 36 beams with either 0.22 or 0.33 cm/deg MLC motion constraint.

*MLC Constraint, cm/deg*	*No. of Beams*	*Average RMS error, cm*	*Max RMS Error, cm*	*% Errors* <0.35 cm	*Max leaf deviation range, cm*
0.22	18	0.027±0.005	0.031±0.03	100	−0.09 to 0.08
0.33	18	0.039±0.01	0.048±0.05	100	−0.20 to 0.18

#### C.5 SmartArc – overall dosimetric agreement

Dosimetric results for 28 plans based on the four structure sets and objectives described in Methods are presented in Table 6. Both ion chamber and gamma analysis results are acceptable for the prostate and multi‐target cases. For the more complex H&N and C‐Shape cases, the gamma analysis results are favorable for the dual‐arc plans.

**Table 6:** Dosimetric analysis of 28 plans for four structure sets. All plans calculated with 2° CP spacing on the 2 mm grid.

*Plan*	*N*	*Approximate Dose Rate Range (MU/min)*	*Mean IC Error PTV, %*	*Mean IC Error OAR, %*	*Mean IC error PTV & OAR, %*	Mean %γ(3%,3mm)≤1.0
Mock Prostate	4	230	0.3	1.8	1.1±1.3	100
Multi‐Target	6	380–450	−1.7	−0.3	−0.7±2.9	97.4±0.8
Mock H&N, 1 arc	6	550–580	−4.8	−1.05	−3.0±2.7	95.2±1.6
Mock H&N, 2 arcs	4	210–460	−3.9	−0.75	−2.3±1.82	98.4±1.1
Mock H&N combined	10	‐	−4.5	−0.9	−2.7±2.3	96.5±2.1
C‐Shape, 1 arc	4	800	−0.6	−5.2	−2.9±3.2	91.4±1.4
C‐Shape, 2 arcs	4	160–350	−0.2	−2.3	−1.3±1.6	98.7±0.4
C‐Shape combined	8	‐	−0.4	−3.7	−2.1±2.6	95.0±3.8

For the C‐Shape, the overall ion chamber point dose error is under the preferred 1.5% threshold.^(^ ^26^ ^)^ The 2.3% overall error for the double‐arc H&N plans is still within the more relaxed fall‐back 3% threshold,^(^ ^26^ ^)^ but it merits further discussion. With the single‐arc plans, both the gamma analysis and point‐dose error results deteriorate for the two more complex plans (Table 6).

#### C.6 SmartArc delivery time

For all plans except C‐shape, the delivery time per arc was 79–80 sec. Two C‐shape plans that were planned with the highest delivery time (150 sec specified, 150–160 sec estimated), required the largest number of monitor units (1300–1500), which resulted in higher actual treatment times of 101 and 115 sec for the MLC constraints of 0.22 and 0.33 cm/deg, respectively.

## IV. DISCUSSION

### A. Leaf‐end radius

The Pinnacle system representation of the curved leaf end was designed to model the standard Varian MLC. The rounded leaf tip radius and the leaf position offset are used to generate the increase in transmission in transition from the full leaf thickness to the tip.^(^ ^11^ ^)^ Geometrically, the leaf tip radius for a standard Varian MLC is close to 8 cm. However, both Cadman et al.^(^ ^11^ ^)^ and, later, Williams and Metcalfe^(^ ^22^ ^)^ reported the best dosimetric agreement achieved with R=12 cm. This is a 50% difference from the best geometrical fit. The shape of the BrainLab mMLC leaf end does not lend itself easily to an approximation by a segment of a circle (Fig. 1). Therefore, the 28 cm value derived from Eq. (1) was not expected to be anything more than a rough starting point. The best dosimetrically determined value of 20 cm is within 30% of the geometrical approximation. The calculated dose profile for R=20 cm is within 1.3% (normalized in the middle of the open field −1.5 cm from the match line) of the measured one for all the points except those within 2 mm of the junction line. The error increases from −3% at 2 mm away from the central axis to −8% at the match line. The error at the junction line is in the same direction as reported for the standard MLC (−5%).^(^ ^22^ ^)^ Although the error is significant at face value, it has to be put in perspective with respect to the discussion by Williams and Metcalfe^(^ ^22^ ^)^ – a 0.1 mm change in MLC calibration leads to 10% change in the match line dose. The static tests are capable of detecting the MLC leaf position deviations of above 0.2 mm.^(^ ^20^ ^)^ In the dynamic delivery situation, the random errors in the leaf position are an order of magnitude larger (Table 5). We have to conclude, following Williams and Metcalfe, that the match line dosimetry is as accurate as it realistically can be.

### B. Table attenuation

The table attenuation in our case is higher than reported previously^(^ ^12^ ^,^ ^23^ ^)^ because of the lower energy of the SRS beam employed in this study, compared to the standard Clinac 6 MV beams. Despite the simple representation of the couch by a single contour with an empirically adjusted density, agreement between measured and calculated attenuation coefficients is quite good (maximum deviation for a single incidence angle of 1.1% for a $10\times 10\;{\text{cm}}^{2}$ field). While the maximum error is slightly higher than that reported by Mihaylov et al.^(^ ^23^ ^)^ (0.5% for 6MV), this is a second‐order difference that will have negligible effect on the multi‐beam treatment plans. Given the excellent average agreement between the measured and calculated doses presented in the Results section, it is justified to use our simple model in clinical routine.

### C. Leaf speed

Our Dynalog file analysis is in line with Ling et al.,^(^ ^29^ ^)^ demonstrating that during the dynamic arc delivery the deviation between the actual and intended leaf positions increases with leaf speed. In the dynamic arc mode, the beam cannot be held off if the leaves did not reach their intended positions because that would involve stopping and starting the gantry rotation. The leaf position interlock is set to a high value (typically 1 cm), again to avoid frequent gantry rotation interruptions. It is up to the user to assure that the leaf position deviations do not lead to unacceptable dosimetric errors. We have previously demonstrated that when the Millennium MLC meets Varian dynamic arc specifications, excellent dosimetric results are achieved with the SmartArc plans delivered on a fully VMAT licensed Trilogy accelerator.^(^ ^27^ ^)^ Since the optimization options in this work are already limited by the inability to vary the gantry speed and dose rate, it would be beneficial to provide the optimizer with greater flexibility in terms of the leaf speed. At least one of the plans had a better PTV coverage with the 1.5 cm/sec maximum leaf speed compared to 1.0 cm/sec (Fig. 3(b)). The Dynalog results for all plans constrained to 1.5 cm/sec maximum leaf speed are within the manufacturer's specifications, while no improvement in dosimetric agreement is seen by limiting the leaf speed to 1 cm/sec (Table 2). We conclude that the optimal leaf speed constraint is the published value of 1.5 cm/sec (0.33 cm/deg), as opposed to the Varian dynamic arc acceptance test^(^ ^30^ ^)^ value of 1 cm/sec.

### D. DMPO planning results and dosimetric agreement

The only significant difference between DMPO planning for the AAPM TG119 structure sets with the mMLC and the Millennium MLC is the inability to achieve the dose‐volume objectives for the multi‐target (stacked cylinders) plan (Fig. 3(b)). This is not a function of the MLC or software design but rather a matter of changing balance between the target dimensions and dose‐volume objectives, since we had to shrink the targets. Inability to meet the dose‐volume objectives in this one case is irrelevant to the overall dosimetric accuracy evaluation. Both the ion chamber and Delta4 dosimetric agreement for DMPO IMRT compare favorably with the excellent results we reported before with the Millennium MLC.^(^ ^4^ ^)^

### E. SmartArc dosimetry

#### E.1 Planning

**Figure 4:**
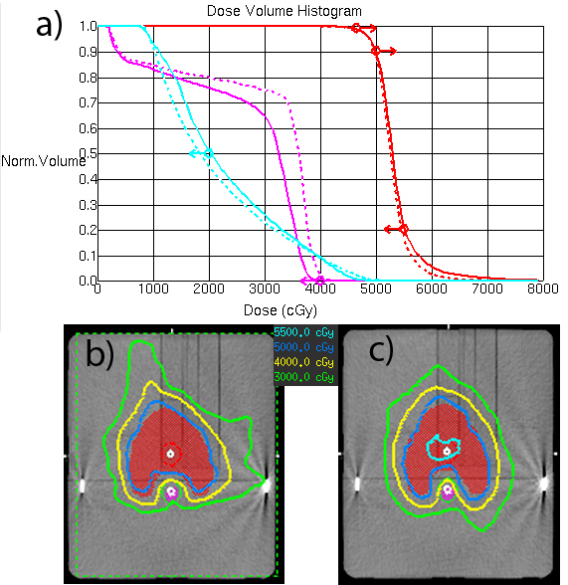
Mock H&N SmartArc plan comparison between single and double‐arcs: a) DVH comparison; b) isodoses for the single‐arc plan; c) isodoses for the double‐arc plan. Solid=single‐arc, dashed=double‐arc plan.

Dose‐volume objectives were easily met for the prostate case (Fig. 3(a)). The multi‐target case objectives were not quite met for the same reason as with the DMPO plan. The SmartArc converges to a slightly different compromise solution (Fig. 3(b)). In the H&N case, all the objectives are met only by the double‐arc plan (Fig. 4). Both plans in that Figure constrained the MLC movement to 1.5 cm/sec (0.33 cm/deg). One has to be cautious comparing treatment plans in terms of dosimetric results not tied to the specified objectives. However, it is apparent from Fig. 4 that double‐arc optimization naturally tends to produce a more conformal dose distribution.

The C‐shape target and OAR geometry at the central axis are similar to H&N, except the PTV volume is smaller and the OAR is closer to it. The planning results are also similar in that only the double‐arc plan meets the objectives, and also naturally produces a more conformal dose distribution. The Novalis planning results for H&N and C‐shape are similar to the previously reported^(^ ^27^ ^)^ Trilogy plans – it takes two arcs to fully satisfy the dose‐volume objectives. The ability to vary the dose rate/gantry speed on the Trilogy does not provide a visible advantage in that regard for those two sets of plans.

#### E.2 Effect of calculation grid size on dosimetric agreement

The results presented in this paper for the Novalis system (Table 3) are different from the previous report on the Trilogy accelerator with the Millennium MLC.^(^ ^27^ ^)^ In the latter case, no meaningful difference in calculated dose distributions was observed while changing the calculation grid voxel size from 3 to 2 mm. The minimum Millennium MLC leaf width is 5 mm, which makes the calculation results relatively insensitive to such change. On the other hand, the smallest mMLC leaf width is 3 mm, which explains the difference between the 3 and 2 mm dose grid. It is prudent to use the 2 mm grid size for calculating SmartArc plans with the mMLC. This is not the case for the DMPO plans, where the minimum number of adjacent leaves creating an opening can be set to two or more.

#### E.3 Overall dosimetric agreement

The results discussed below were obtained with the most accurate practical dose calculation parameters: 2 mm grid size and 2° CP spacing. The previous Trilogy results^(^ ^27^ ^)^ used for comparison were obtained with the full VMAT capabilities. Only plans optimized with the 2° CP spacing are compared.

The prostate case involves limited MLC motion. Dosimetric agreement is excellent (Table 6) for both ion chamber and Delta4 measurements. The magnitude of the point dose errors is similar to the Trilogy. The γ(3%,3mm) passing rate is 100% for both machines.

The mean multi‐target point dose error is within 1% for the Trilogy and within 2% for the Novalis when calculated separately for each target. When the results for all the cylinders are combined, the mean point dose error for the Novalis is also under 1%. The γ(3%,3mm) passing rate is slightly lower for the Novalis (97% vs. 100%).

The results are more interesting for the more complex plans – H&N and C‐shape. The Novalis single‐ and double‐arc plans can be clearly segregated in terms of the gamma analysis passing rates. The average γ(3%,3mm) passing rate is lower for the single‐arc H&N plans compared to the double‐arc ones (95% vs. 98%), but particularly so for the C‐Shape plans (91% vs. 99%). The relative dose‐error distribution for the double‐arc plans clearly peaks more strongly around zero than the single‐arc ones. A similar but less pronounced trend was observed with the Trilogy plans, where the gamma passing rate difference between the single‐ and double‐arc plans was limited to 2–3 percentage points.

At face value, the Novalis ion chamber point dose errors for the complex cases seem quite large (Table 6), particularly when compared to the Trilogy results where the mean deviations did not exceed 1.1%. However, a closer look reveals a pattern: the mean errors are large in the PTV for the H&N and in the OAR for the C‐Shape. Conversely, the mean error does not exceed 1.1% for the H&N OAR and the C‐Shape PTV. The structure sets on the central transverse slice are similar in general shape. The difference is the position of the contours in relation to the measurement points. For the H&N, the PTV measurement point is at the isocenter, and so is the OAR point for the C‐Shape. The dose errors are larger around the isocenter and smaller elsewhere.

**Figure 5:**
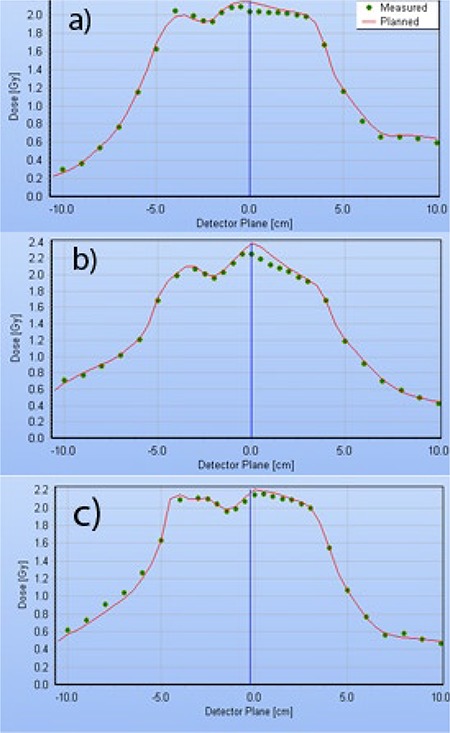
Delta4 dose profiles taken in the transverse plane, through isocenter, at a 50° angle, corresponding to the Delta4 main detector board orientation. H&N plans constrained to 0.33 cm/deg MLC movement: (a) single‐arc; (b) double‐arc with individual beams ALPO 4.8 and 1.7 cm; (c) double‐arc with individual beams ALPO 3.8 and 2.3 cm.

Although the dose profiles perpendicular to the leaf movement can have large dose gradients (up to 5%/mm) because of the narrow MLC leaves, the dose error at the isocenter is persistent and cannot be remedied significantly by moving the chamber around by a millimeter or two, as previously suggested.^(^ ^26^ ^)^ Typical dose profiles for the H&N single‐ and double‐arc plans are presented in Fig. 5. The relative dose errors for the central diode are within 1.2 percentage points of the ion chamber, which is a good agreement given the differences in the detector and phantom size and type.

To obtain a better representation of the relative dose error, we analyzed the combined statistics from all the Delta4 measurements (Table 7). The standard deviations are relatively large (4%–5%) because the results are reported for all the measurement points, including those in the high dose gradient areas. The mean dose error for all structure sets, except one, is below 1%, and the overall mean error for more than 13,000 measurement points is under 1% as well. This shows that point dose measurements at the isocenter may not be representative of the overall dosimetric agreement for the complex plans in question.

**Table 7:** Mean relative dose‐errors measured with Delta4.

	*Relative Dose‐error, %*						
*Plan*	*Prostate* (N=1956)	*Multi‐Target* (N=3388)	*H&N Single‐arc* (N=3371)	*HN Double‐arc* (N=2330)	*C‐Shape Single‐arc* (N=1507)	*C‐Shape Double‐arc* (N=1938)	*All* (N=13710)
	0.1±2.5	−1.6±4.2	−0.7±4.4	−0.8±4.1	−2.3±5.0	−0.9±4.6	−1.0±4.2

When the accuracy of open field dosimetry has been proven across the range of field sizes, the usual suspect for the excessive IMRT dose errors is MLC transmission.^(^ ^26^ ^)^ Although there is a small (−1%) overall bias in the dose‐error distribution with the SmartArc plans, at this time we would be reluctant to change the MLC transmission in the model. The commissioning process for the step‐and‐shoot IMRT is much better understood. The DMPO dose errors we observed, including the complex plans, are quite small. This suggests a properly selected MLC transmission value. Also, on average, the measured dose is lower than predicted. To minimize this bias one would have to decrease the MLC transmission. The 0.9% value we use is already the lowest in the range reported in the literature for the BrainLab mMLC (0.9%–1.9%).^(^ ^36^ ^)^

**Figure 6:**
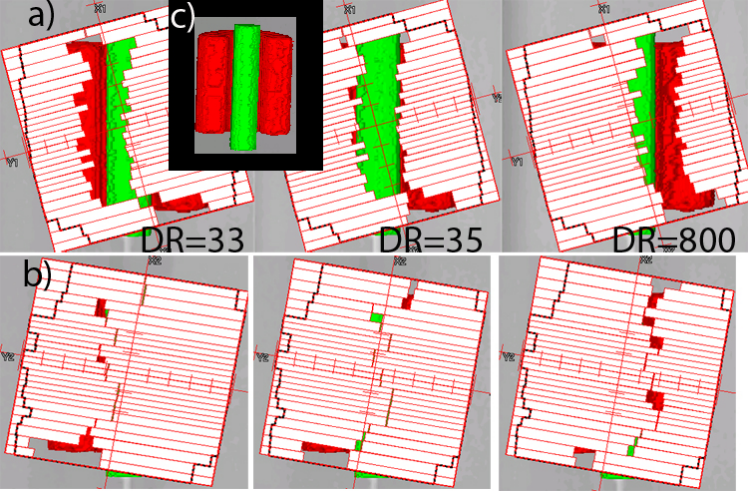
C‐Shape single‐arc plans with MLC apertures for control points separated by 4°: (a) variable dose rate (DR) plan on a hypothetical linac; (b) constant dose rate plan, real Novalis linac; Insert (c) unobscured posterior view of the structure set.

Since we did not observe dose errors preferentially gravitating towards the central axis with the fully VMAT‐capable linac,^(^ ^27^ ^)^ for the same structure sets, it was instructive to compare the optimization solutions. A hypothetical linac was created in Pinnacle. It had all the parameters of the actual Novalis machine, except that dose rate and gantry speed variation were allowed for SmartArc planning. Two C‐shape plans were optimized with the identical objectives but using these different linac models. Although the solutions lead to very similar DVH curves, they were markedly different in terms of the MLC apertures. Figure 6 shows three control points separated by 4° each, for the hypothetical VMAT linac (a) and the real one (b). The beam directions are close to posterior. The optimizer needs to find a way to irradiate a portion of the PTV (red) on the left, transition through the middle while sparing the OAR (green), and proceed with irradiating the remainder of the target on the right. For the VMAT linac, the optimizer maintains a sizable MLC opening, but drops the dose rate from 800 MU/min to the minimum allowed by the SmartArc software −30 MU/min. With the real linac operating at a constant dose rate (800 MU/min in this case), the optimizer has no choice but to close the MLC (Fig. 6(b)). This effectively results in the very small gaps (0.5 mm) between the leaves traveling across the field while the monitor units are being delivered at the fastest possible rate. This is an overly challenging situation for any dose calculation algorithm.

The difference in the MLC apertures is reflected in the average leaf pair openings (ALPO)^(^ ^39^ ^)^ for the similar C‐shape plans: 1.3 cm for the single‐arc realistic Novalis plan vs. 2.3 cm for the hypothetical VMAT‐capable linac. Double‐arc C‐shape plans largely avoid the leaf tips crossing the central axis, resulting in better dosimetric agreement at the isocenter (Table 7). It is also instructive to compare two different double‐arc H&N plans represented in Figs. 5(b) and (c). During optimization, the plans were allowed different delivery times per arc (80 vs. 90 sec), and the optimizer arrived at two different solutions. In both cases, one of the two arcs has a larger average opening, but the difference varies (ALPO 4.8 and 1.7 cm in (b), and 3.8 and 2.3 cm in (c)). Visual inspection revealed that the 1.7 cm ALPO arc, corresponding to Fig. 5(b), had a significant number of MLC apertures similar to Fig. 6(b), with the resulting dose disagreement in the center. On the other hand, both arcs corresponding to Fig. 5(c) largely avoid control points with extremely narrow openings, and dosimetric agreement in the center is substantially better. We believe there are enough data presented to suggest that the extremely narrow MLC openings traveling across the field while the monitor units are being delivered at a high rate are responsible, at least in large part, for the dosimetric errors not previously seen with the VMAT‐licensed Trilogy linacs.

When only the double‐arc plans are used for the complex cases, the largest mean ion chamber dose error for an individual case (H&N) is −2.3%, with the other three cases exhibiting less than 1.5% deviations. Averaged across all four cases, the mean error is −0.8%. Again, including only the double‐arc plans for the complex cases, the mean γ(3%,3mm) passing rates for individual cases are in excess of 97%, with the average across all four cases being 98.6%. It is essentially the same number as previously measured with the Delta4 for the SmartArc plans on the VMAT‐enabled Trilogy linac (98.2%)^(^ ^27^ ^)^ or for RapidArc treatments (98.5%),^(^ ^40^ ^)^ both of which were characterized as clinically acceptable. Based on our results, it is advantageous, in terms of the dosimetric accuracy, to employ the double‐arc plans for the more complex cases.

## V. CONCLUSIONS

For an institution that already owns the licenses, it is economically prudent and technically feasible to use the Pinnacle TPS with the BrainLab Novalis delivery system. This allows taking advantage of the improved accuracy of the CCC algorithm in the presence of heterogeneities, compared to the pencil beam calculations. The reference patient positioning DRRs still have to be generated by the BrainLab software, from the CT images and the isocenter coordinates transferred from Pinnacle. We validated this process with the end‐to‐end hidden target test. The Novalis treatment table attenuation is substantial and needs to be accounted for in calculations. The attenuation is higher for the high dose rate SRS beam than for the previously reported standard Varian Clinac 6 MV beams. A simple single‐contour treatment table attenuation model is sufficiently accurate for routine clinical use. The mMLC leaf tip, although not geometrically round, can be represented in Pinnacle by an arch with satisfactory dosimetric accuracy. Subsequently, step‐and‐shoot (DMPO) dosimetric agreement is excellent. VMAT (SmartArc) treatments with constant gantry speed and dose rate are feasible without any modifications to the accelerator. For the more complex cases, dual‐arc SmartArc plans lead to better results both in terms of meeting the dose‐volume objectives and dosimetric accuracy. Unlike the previously investigated SmartArc plans with the Millennium 120 leaf MLC (minimum leaf width 5 mm), with the mMLC (3 mm leaf width), dose distributions do change when the calculation grid is reduced from 3 to 2 mm. The use of a 2 mm grid is, therefore, recommended. When double‐arc plans are used for the more complex plans, the overall dosimetric agreement for the SmartArc on a Novalis linac compares favorably with the previously reported results for other implementations of VMAT. However, a larger than previously observed dose error with the single‐arc plans, confined predominantly to the isocenter region, requires further investigation.
